# Impact of body mass index on hemoglobin level and blood transfusion in total knee arthroplasty: A retrospective case control study

**DOI:** 10.1016/j.amsu.2020.05.028

**Published:** 2020-05-28

**Authors:** Khaldoon Bashaireh, Osama Aljararhih, Khaldoon Alawneh

**Affiliations:** a Jordan University of Science and Technology (JUST), Department of Special Surgery, College of Medicine, Irbid, Jordan. P.O.Box 3030, Irbid, 22110, Jordan; b Jordan University of Science and Technology (JUST), Department of Medicine, College of Medicine, Irbid, Jordan. P.O.Box 3030, Irbid, 22110, Jordan

**Keywords:** Total knee arthroplasty, Obesity, Blood transfusion, Body mass index, BMI, body mass index, Hb, hemoglobin, TKA, total knee arthroplasty

## Abstract

**Background:**

Morbid obesity is a challenge in cases that require total knee arthroplasty, and several studies considered it a contraindication for the procedure due to associated risk of complications, including plummeting hemoglobin levels and subsequent need for a blood transfusion. This study investigated risk factors for blood transfusion in this patient group and considered their relationship to obesity.

**Materials and methods:**

Patients’ data were extracted from medical records, including estimated blood loss and perioperative hemoglobin levels. Patients were weighed and measured, and their body mass index (BMI) was calculated and stratified according to international criteria.

**Results:**

A total of 188 patients were included in this study; among them, 136 patients had obesity (72%), with a mean BMI of 33.54. The mean volume of blood lost was 1055.4 ml, with the mean postoperative hemoglobin decrease of 1.42 g/dl and 2.88 g/dl at 6 and 24 h after surgery, respectively. The pre-operative Hb level was the only significant risk factor for blood transfusion. BMI did not affect the risk of blood transfusion or amount of blood lost.

**Conclusion:**

Obesity (BMI > 30 kg/m^2^) did not increase the risk of needing a blood transfusion after total knee arthroplasty. A judicious transfusion strategy involving the pre-operative Hb optimization should be adopted in TKA to decrease transfusion rate, benefit patient outcomes, and increase healthcare system efficiency. This study shows that high BMI is not a risk factor for postoperative blood transfusion.

## Highlights

• Obesity is a challenge for total arthroplasty due to risk of complications.
• Need of blood transfusion are common among obese patients post-surgically.
• Whether obesity increased the risk of needing a blood transfusion is not clear.
• In this study, obesity did not increase the risk of needing blood transfusion.
• Pre-operative Hemoglobin level was a risk factor for needing blood transfusion.

## 1 Introduction

Total knee arthroplasty (TKA) is a common surgical procedure recommended for patients with severe knee osteoarthritis. Patients who undergo this surgery report abated pain, improved functional scores, and satisfactory quality of life [1]. Given these benefits, understanding risk factors for complications associated with this surgery is important.

Morbid obesity is considered a contraindication for total joint arthroplasty [2]. Obesity is a worldwide public health concern, as the number of people with obesity continues to increase, significantly affecting population health. Obesity is a risk factor for osteoarthritis, which may require TKA; concurrently, obesity increases the risk of osteoarthritis in younger age groups, making young people candidates for surgery [3].

In Jordan, the number of people with obesity has incrementally increased between 2004 and 2019. The prevalence of obesity in Jordan is expected to be 34.3%, which would put Jordan directly behind the United States, where the prevalence is estimated at 35% [3,4].

The definition of obesity based on body weight is commonly used in clinical practice and research, including at our institution. Body mass index (BMI) is considered a reliable indicator of risks associated with excessive weight; it relates well to body fat percentage [5].

Previous studies have reported on perioperative difficulties and complications associated with knee arthroplasty in obese patients [6,7]. These studies have highlighted the influence of obesity on the need for blood transfusion; however, this association remains controversial [[8], [9], [10], [11], [12], [13], [14], [15]]. Blood transfusion itself carries risks, such as infections, and should be pursued with caution [16]. Different strategies for perioperative blood management are available and can be used in numerous combinations; adoption of these strategies may improve outcomes and decrease the cost of TKA, in particular, if strategies are differentiated based on patients’ specific risk factors, expected blood loss, and comorbidities, which are factors considered at different centers [17]. The majority of previous studies on perioperative complications associated with knee arthroplasty among obese patients are retrospective [3]. This study investigated the impact of BMI on perioperative hemoglobin (Hb) level and risk of blood transfusion in patients undergoing total knee arthroplasty.

## 2 Materials and methods

This retrospective study involved paper-based and electronic medical records of 188 consecutive patients admitted to a tertiary academic center and who underwent primary TKA. Patients were eligible for inclusion if they were undergoing primary TKA due to severe osteoarthritis. Patients were excluded from this study if they were undergoing a revision surgery, had a blood disease i.e. hemophilia, were receiving anticoagulants, or developed anemia prior to surgery.

All surgeries were performed by the same surgical team, who followed a standard procedure. Standard medial parapatellar arthrotomy was used in all surgeries. A pneumatic tourniquet was applied in 81 patients before skin incision and it was released after cementing the prosthesis; the femoral and tibial components were cemented; the patellas were not resurfaced. Two types of prosthesis were used in this study: NexGen® (Zimmer Biomet, Ltd., Swindon, UK) and PFC Sigma PS fixed (DePuy Synthes Inc. Warsaw, IN, USA). Patients were admitted within 1–2 days prior to surgery and discharged 5 days post-surgery. Pre- and post-operatively, patients were treated according to the institution specific clinical practice guidelines for TKA. Recommended thromboprophylaxis, consisting of chemical (daily low-molecular-weight heparin 40 mg SC) and mechanical (intermittent pneumatic compression devices) prophylaxis was used for each patient. A closed drainage system was used routinely and removed 48 h post-surgery. All patients underwent a standard unified rehabilitation program following surgery [18].

The study was approved by an ethical committee and conducted according to the principles of the Declaration of Helsinki (approval number withheld for peer review). The written consent requirement was waived due to the retrospective nature of the study. All data were confidential. The study was conducted in line with the STROCSS criteria [19]. Also, the article was registered with the UIN: researchregistry5479.

Estimated perioperative blood loss in milliliters (ml) was calculated based on the hemoglobin dilution formula [20]. The volume of blood loss = blood volume *(Hb before surgery−Hb after surgery)/Hb before surgery [20]. Blood volume = k1*height^3+k2*weight + k3, in which k1 = 0.3669, k2 = 0.03219, and k3 = 0.6041 in males, while for females, k1 = 0.3561, k2 = 0.03308, and k3 = 0.1833 [19]. The following demographic characteristics were extracted from patients’ medical records: age, sex, primary diagnosis, comorbidities, side of surgery, type of anesthesia, type of prosthesis, duration of hospital stay, estimated blood loss (ml), perioperative Hb levels in grams (g) per deciliter B (dL), BMI (BMI = body weight [kg]/height-squared [m^2^]), and number of transfused blood units. Data collection was performed by the same team for all patients. All patients were followed for 6 weeks postoperative. All patients were compliant with the postoperative instructions.

Patients who met inhouse transfusion criteria were transfused with allogenic blood. Indications for transfusion included Hb levels either below 8 g/dl (transfusion definitely required) or between 8 and 10 g/dl with concurrent symptoms or presence of heart disease or another major cardiovascular risk. Blood transfusion did not affect the duration of hospital stay.

Statistical analyses were performed using IBM SPSS Statistics Software (v.21), 2012 (IBM, Armonk, New York, USA). Data were presented as frequency distributions for categorical variables (presence of obesity, number of blood unit transfused, type of anesthesia, diagnosis, and use of tourniquet) and mean ± standard error of the mean for continuous variables (age, blood loss, and BMI). Pearson χ^2^ test was used to investigate the significance of association between categorical variables, while unpaired student's *t*-test and ANOVA were applied to examine the significance level for continuous normally distributed variables. Multivariate logistic regression analysis was used to examine the effect of each variable on blood transfusion rate; odds ratios (OR) and confidence intervals (CI) were calculated and reported alongside corresponding p-values. A p-value <0.05 was considered indicative of a statistically significant finding.

## 3 Results

### 3.1 Sample characteristics

Data from a total of 188 patients were included in this retrospective study. The mean age of the included patients was 64.13 years (SD = 7.24) (range, 46–82). The mean admission duration was 8.78 days (SD = 1.49) (range, 5–13). The mean BMI was 33.54 (SD = 4.72) (range, 23.18–46.10). Based on the BMI criteria, 136 patients were obese (72%). The mean Hb loss at 6 and 24 h after surgery was 1.40 g/dl (SD = 0.82) and 2.85 g/dl (SD = 1.37), respectively. The mean amount of blood lost was 1055.4 ml (SD = 467.3) (range, 40.9–2262.2 ml). The mean preoperative Hb was 12.6 g/dl. In addition, 108 patients (57.40%) required no blood transfusion, while 26 (13.80%), 47 (25.00%), and 7 patients (3.70%) required 1, 2, and 3 units of blood, respectively.

Over half of all surgical procedures (53.20%) were performed on the right side, and the majority of these procedures were performed under general anesthesia (87.20%). Only 24 surgeries (12.80%) were performed under spinal anesthesia. Tourniquet was used in 81 patients (43.1%). Two-thirds of the included patients had comorbid diseases. The majority of the included patients were diagnosed with osteoarthritis, while only 20 patients (10.60%) were diagnosed with rheumatoid arthritis. These findings are summarized in Table 1.

**Table 1:** Clinical and demographic characteristics of patients undergoing arthroplasty surgery (N = 188).

Variables	Range	Mean (SD)	Count	Percent %
Age (Years)	46.00–82.00	64.13 (7.24)		
Admission duration (Days)	05.00–13.00	08.78 (1.49)		
Body mass index	23.18–46.10	33.54 (4.72)		
Amount of blood loss	40.9–2262.2	1055.4 (467.3)		
Preoperative Hb	9.0–16.0	12.6 (1.2)		
Hb loss at 6 h after surgery	0–3.60	1.42 (0.82)		
Hb loss at 24 h after surgery	0–6.50	2.88 (1.37)		
Blood transfusion				
Yes			80	43.60
No			108	57.40
Obesity				
Yes			136	72.30
No			52	27.70
Site of surgery				
Right			88	46.80
Left			100	53.20
Type of anesthesia				
General			164	87.20
Spinal			24	12.80
Using tourniquet				
Yes			81	43.10
No			107	56.90
Diagnosis				
Osteoarthritis			168	89.40
Rheumatoid arthritis			20	10.60
Comorbid diseases				
Yes			121	64.40
No			67	35.60

Hb, hemoglobin.

### 3.2 Factors affecting the need for blood transfusion

Patient age, obesity status, surgery site, type of anesthesia used, use of tourniquet, or presence of co-morbidities did not influence the need for blood transfusion. In contrast, preoperative Hb, volume of blood loss and Hb loss at 6 and 24 h affected the need for blood transfusion (Table 2).

**Table 2:** Analysis of pre- and post-operative factors associated with the need for blood transfusion (using Pearson χ^2^ and *t*-test).

Pre- and post-operative Variables	Transfusion N (%)	No transfusion N (%)	*P*-value
**Sex**			NS
Male	7 (8.8)	8 (7.4)	
Female	73 (91.2)	100 (92.6)	
**Age (years)**	64.5 ± 0.8	63.5 ± 0.7	NS
**Comorbidities**	48 (60.0)	73 (67.6)	NS
**Obesity**	53 (66.3)	83 (76.9)	NS
**Site**			
Right	33 (41.3)	55 (58.7)	NS
Left	47 (50.9)	53 (49.1)	
**Type of anesthesia**			NS
General anesthesia	73 (91.2)	91 (84.3)	
Spinal anesthesia	7 (8.8)	17 (15.7)	
**Tourniquet use**	37 (46.3)	44 (40.7)	NS
**Diagnosis**			NS
Osteoarthritis	69 (86.2)	99 (91.7)	
Rheumatoid arthritis	11 (13.8)	9 (8.3)	
**Admission duration (days)**	9.2 ± 0.2	8.9 ± 0.2	NS
**Preoperative Hb g/dl**	12.9 ± 0.1	12.1 ± 0.1	0.000
**Blood loss (ml)**	1276.7 ± 59.1	898.2 ± 36.4	0.000
**Hb loss at 6 h (g/dl)**	1.7 ± 0.1	1.3 ± 0.1	0.006
**Hb loss at 24 h (g/dl)**	1.8 ± 0.2	1.4 ± 0.1	0.000
**BMI (kg/m**^**2**^**)**	33.0 ± 0.6	34.1 ± 0.4	NS

Hb, hemoglobin.

Logistic regression included the following independent variables: admission duration, age, Hb loss at 6 and at 24 h after the surgery, BMI, obesity, site of surgery, type of anesthesia, use of tourniquet, amount of blood in the drain, diagnosis, and comorbidity.

The model that included all variables was robust at ascertaining blood transfusion need (X^2^ [12] = 61.1, P = 0.000). Moreover, Negelkerke R^2^ was 0.38, indicating the strength of association between the predictors and the odds of blood transfusion need.

The regression revealed preoperative Hb as the only significant predictor of blood transfusion need. The OR for preoperative Hb was 0.3. No other variable was a statistically significant predictor of transfusion need in the logistic regression model (Table 3).

**Table 3:** Results of logistic regression analysis showing variables affecting the risk of needing blood transfusion among patients undergoing arthroplasty surgery (N = 188).

Variable	B	Wald	P-value	OR	CI
Age	0.01	0.13	0.71	1.01	0.96–1.05
Admission duration	0.18	2.82	0.28	1.20	0.96–1.49
Hb loss at 6 h after surgery	−0.18	0.52	0.19	0.83	0.50–1.37
Hb loss at 24 h after surgery	0.53	1.1	0.28	1.8*	1.24–2.32
**Preoperative Hb**	**−1.1**	**18.3**	**0.000**	**0.3**	**0.19–0.46**
Body mass index	−0.05	1.06	0.30	0.95	0.85–1.05
Obesity	0.26	0.22	0.63	1.30	0.45–3.73
Site of surgery	−0.32	0.96	0.33	0.72	0.38–1.38
Type of anesthesia	0.47	0.83	0.36	1.60	0.58–4.20
Using tourniquet	−0.27	0.64	0.42	0.76	0.39–1.47
Volume of blood loss	000	0.002	0.001	0.67	0.99–1.00
Diagnosis	−0.30	0.30	0.58	0.74	0.25–2.15
Comorbid diseases	0.35	1.00	0.31	1.42	0.71–2.82

OR, odds ratio; CI, confidence interval; Hb, hemoglobin.

Findings were considered significant at p < 0.05.

### 3.3 Factors affecting Hb loss at 24 h

**Fig. 1:**
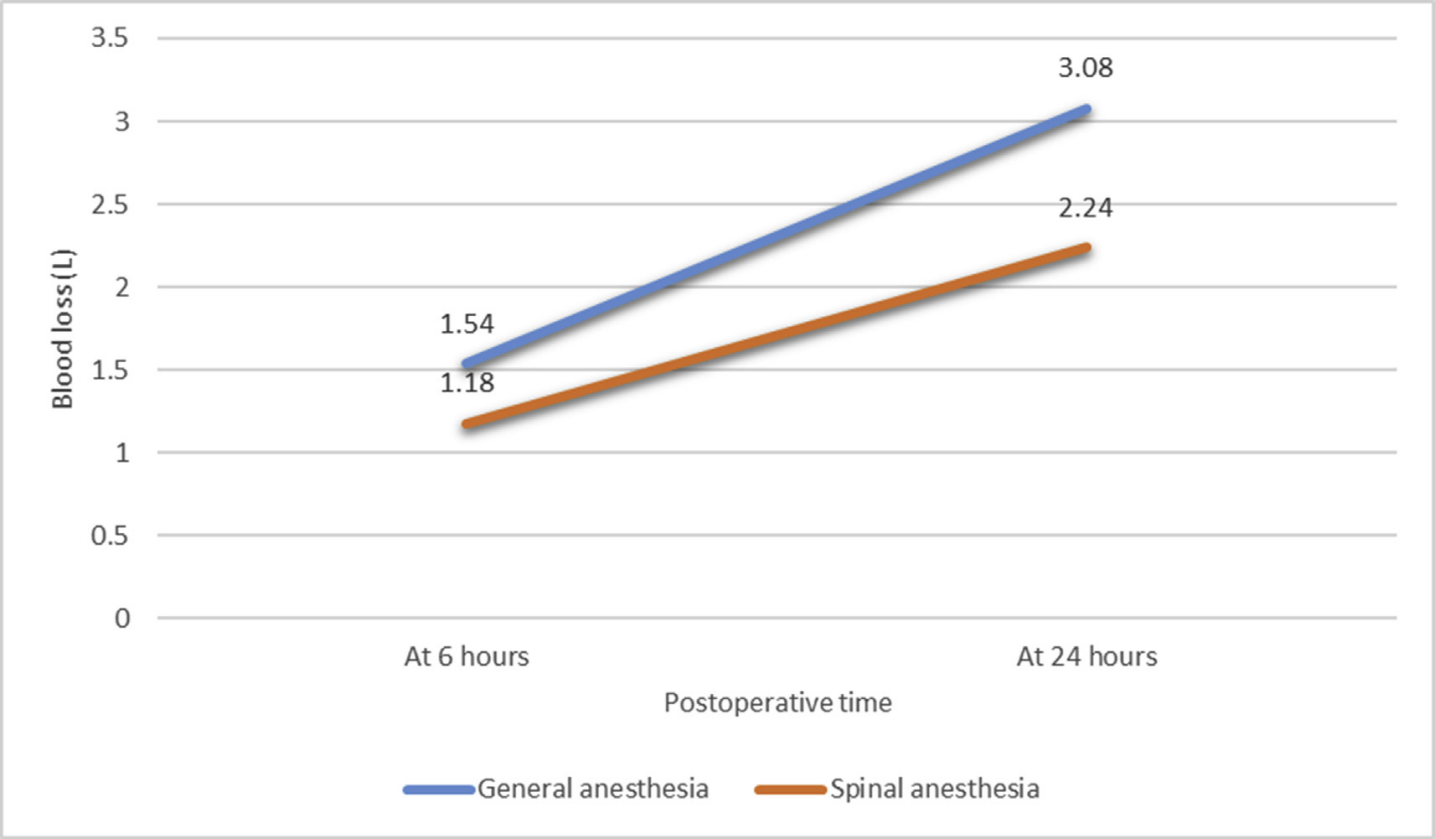
legend: Chart indicating the relation between Hb loss and type of anesthesia.

The only factor to increase the amount of Hb lost at 24 h post-surgery was the type of anesthesia used. Following general anesthesia, the mean Hb loss at 24 h was 1.67 g/dl; in contrast, following spinal anesthesia, it was 1.25 g/dl (P = 0.018). There was no effect of BMI or obesity on the need for blood transfusion Fig. 1 chart showing the relationship between type of anesthesia and blood loss.

## 4 Discussion

Obesity is a risk factor for various diseases, and its impact on general health is of interest worldwide [21]. Patients with obesity are at a higher risk of osteoarthritis, and more likely to require replacement of hips and knees [22], compared to their non-obese counterparts. These patients are also more likely to experience complications post-surgery. This study aimed to identify factors that influence the need for blood transfusion among patients with obesity and verify whether obesity is an independent risk factor for blood transfusion after TKA.

Different studies highlighted the correlation between increase in BMI and the risk of blood transfusion post-TKA; however, the results of these studies were inconsistent and controversial. For example, Frisch et al. stated that increased BMI corresponded to a significant decrease in the risk of perioperative blood transfusion, suggesting this association to result from a greater basal blood volume in patients with high BMI; other authors have shown similar findings [11,[23], [24], [25]]. Conversely, Noticewala et al. showed that BMI >30 kg/m^2^ was associated with a greater amount of perioperative blood loss and increased risk of blood transfusion [[26], [27], [28]]. In the present study, there was no association between BMI and the increase or decrease in the risk of blood transfusion.

Blood loss is one of the most common side effects of TKA; however, the exact amount of blood lost may vary depending on gender, age, recent use of anticoagulants, and tourniquet use, among others [29]. Accordingly, perioperative transfusion rates vary, with some reporting transfusion rates after TKA as 3%–67% [11]. A blood management program for TKA aims to reduce allogenic transfusion rate and its associated risks [17]. The incidence of transfusion in our study was 42.5%. The relatively high rate of transfusion can be explained by not optimizing the pre-operative Hb level, not using any type of local or intravenous medication i.e. tranexamic acid, and routine use of intra-articular wound drainage in TKA, all of which have been shown to increase the risk of blood transfusion.

Our study demonstrated that estimated blood loss was less among patients with Neuraxial (NA) vs general anesthesia (GA). Walker et al. studied postoperative outcomes associated with NA vs GA following bilateral total knee arthroplasty and showed the same result [30]. The mechanism of reduced requirement of blood transfusions in the setting of NA is unclear. However, this finding may be due to the lower arterial and venous blood pressure induced by NA compared to GA, both intraoperatively and postoperatively (during recovery). Large studies are required to clarify this observation. Additionally, randomized controlled trials are required to validate our findings.

In the present study, the preoperative Hb level was the only significant predictor of transfusion risk regardless of BMI category, which is consistent with the previous studies [31,32]. Patients with a preoperative Hb level <12.1 g/dL are at higher risk of needing a transfusion compared to the other patients. A strategy for optimizing the pre-operative Hb level would help in decreasing the rate of transfusion.

The other variables studied such as age, admission duration, Hb level at 6 h and 24 h after surgery, site of surgery, use of tourniquet, drains readings, diagnosis, or the presence of co-morbidities were not associated with the risk of transfusion.

Although this was a retrospective study, to the best of our knowledge, it is the first study of its kind originating in this region. In addition, as all patients received surgery from the same team, the effect of interobserver variability was reduced, controlling bias.

This study has several limitations. First, it was a retrospective observational study. Second, this study used the definition of obesity as BMI >30, which might be a less accurate representation than percentage body fat. Third, intraoperative blood loss was estimated using the hemoglobin dilution formula which is not the most reliable method of estimating blood loss after TKA. Fourth, a small sample size decreases the power of this study.

## 5 Conclusion

In the present study, obesity (defined as BMI >30) did not affect the amount of perioperative blood loss or blood transfusion rates following TKA. A judicious transfusion strategy involving the pre-operative Hb optimization should be adopted in TKA to decrease transfusion rate, benefit patient outcomes, and increase healthcare system efficiency.

## Provenance and peer review

Not commissioned, externally peer reviewed.

## Ethical approval

Institutional approval was obtained from the Institutional Review Board at Jordan University of Science and Technology (74/128/2019).

## Funding

No funding

## Author contribution

All authors contributed significantly and in agreement with the content of the article. All authors were involved in project design, data collection, analysis, statistical analysis, data interpretation and writing the manuscript. All authors presented substantial contributions to the article and participated of correction and final approval of the version to be submitted.

## Registration of research studies

Researchregistry5479.

## Guarantor

Dr. Khaldoon Bashaireh.

## Funding source

This research did not receive any specific grant from funding agencies in the public, commercial or not-for-profit sectors.

## Declaration of competing interest

The authors declare that they have no competing interests.
